# Evaluation of biotransformation capacity of transplastomic plants and hairy roots of *Nicotiana tabacum* expressing human cytochrome P450 2D6

**DOI:** 10.1007/s11248-022-00305-x

**Published:** 2022-04-13

**Authors:** Y. V. Sheludko, I. M. Gerasymenko, F. J. Herrmann, H. Warzecha

**Affiliations:** 1 Plant Biotechnology and Metabolic Engineering, Technical University of Darmstadt, 64287 Darmstadt, Germany; 2 Centre for Synthetic Biology, Technical University of Darmstadt, 64287 Darmstadt, Germany; 3 Department of Organic Chemistry and Biochemistry, Technical University of Darmstadt, 64287 Darmstadt, Germany

**Keywords:** Plastid transformation, Hairy roots, Cytochrome P450, CYP2D6, Loratadine, *Nicotiana tabacum*

## Abstract

Cytochrome P450 monooxygenases (CYPs) are important tools for regio- and stereoselective oxidation of target molecules or engineering of metabolic pathways. Functional heterologous expression of eukaryotic CYPs is often problematic due to their dependency on the specific redox partner and the necessity of correct association with the membranes for displaying enzymatic activity. Plant hosts offer advantages of accessibility of reducing partners and a choice of membranes to insert heterologous CYPs. For the evaluation of plant systems for efficient CYP expression, we established transplastomic plants and hairy root cultures of *Nicotiana tabacum* carrying the gene encoding human CYP2D6 with broad substrate specificity. The levels of CYP2D6 transcript accumulation and enzymatic activity were estimated and compared with the data of CYP2D6 transient expression in *N. benthamiana*. The relative level of CYP2D6 transcripts in transplastomic plants was 2–3 orders of magnitude higher of that observed after constitutive or transient expression from the nucleus. CYP2D6 expressed in chloroplasts converted exogenous synthetic substrate loratadine without the need for co-expression of the cognate CYP reductase. The loratadine conversion rate in transplastomic plants was comparable to that in *N. benthamiana* plants transiently expressing a chloroplast targeted CYP2D6 from the nucleus, but was lower than the value reported for transiently expressed CYP2D6 with the native endoplasmic reticulum signal-anchor sequence. Hairy roots showed the lowest substrate conversion rate, but demonstrated the ability to release the product into the culture medium. The obtained results illustrate the potential of plant-based expression systems for exploiting the enzymatic activities of eukaryotic CYPs with broad substrate specificities.

**Supplementary Information:**

The online version contains supplementary material available at 10.1007/s11248-022-00305-x.

## Introduction

The cytochrome P450 superfamily comprises enzymes catalyzing a variety of oxidative biotransformations. Cytochrome P450 monooxygenases (CYPs) are enormously variable by their sequences and catalytic activities but maintain a conserved P450 fold (Nelson 2018). Most plant CYPs are specific for one or a limited number of substrates and therefore are responsible for particular conversion steps within a biosynthetic pathway. On the other hand, many animal CYPs display extraordinary catalytic versatility and accept a broad range of substrates (Srejber et al. 2018; Nelson 2018; Schuler 2015; Sezutsu et al. 2013; Schuler and Rupasinghe 2011). In humans, CYPs participating in the metabolism of xenobiotics are predominantly responsible for the detoxication but also the bioactivation of endogenous or foreign compounds (Rendic and Guengerich 2021; Guengerich 2017). These enzymes can convert herbicides and environmental pollutants, as well as numerous plant secondary metabolites used as medicines (Rendic and Guengerich 2015, 2021).With this outstanding catalytic capacity they are regarded as important tools in chemical and biotechnological applications, affording regio- or stereospecific oxidation of target molecules which is hardly achievable via chemical modifications (Li et al. 2020; Guengerich 2018).

Despite the increasing number of applications for native or engineered CYPs as oxidative biocatalysts (Fessner et al. 2021; Li et al. 2020; Fessner 2019), their employment for scaled-up biotransformations is still limited predominantly to soluble CYPs of prokaryotic origin (Wu et al. 2021). Functional heterologous expression of eukaryotic CYPs is often problematic due to their dependency on the specific redox partner and the necessity of correct association with the membranes for displaying enzymatic activity (Srejber et al. 2018; Barnaba et al. 2017). In their catalytic cycle, for oxygen activation CYPs receive two electrons from NADPH. To eukaryotic CYPs the electrons are transported by a membrane-bound flavoprotein, cytochrome P450 reductase (CPR); alternatively, the second electron may be supplied through cytochrome b5 (Srejber et al. 2018; Waskell and Kim 2015). Most eukaryotic CYPs are localized on the endoplasmic reticulum (ER) membrane, although there are examples of CYPs operating in mitochondria (Omura and Gotoh 2017), tonoplasts (Xu et al. 2006) and chloroplasts (Bak et al. 2011). Integration of CYPs into the ER membrane is mediated by an uncleavable N-terminal signal-anchor sequence (Srejber et al. 2018; Guengerich 2015).

Expression of eukaryotic CYPs in *Escherichia coli*, yeasts or mammalian cells often results in poor accumulation and low stability or activity of the enzymes (Wu et al. 2021; Hausjell et al. 2020). In *E. coli*, the expression of enzymatically active native membrane-bound CYPs is inefficient, and successful examples of CYP application for preparative oxidative biotransformation are limited predominantly to the soluble mutants of BM3 CYP from *Bacillus megaterium* (Wu et al. 2021; Hausjell et al. 2018, and references cited therein). Mammalian or insect expression systems provide a cellular environment relevant for eukaryotic CYPs but demand high culture costs and complicated techniques that hampers their utilization for scaled-up enzyme production. Yeasts, possessing similar inner organelles as other eukaryotes, are considered being promising hosts for CYP expression (Hausjell et al. 2020). Recently, Fessner et al. reported 100 mg scale biotransformation of several natural products by human CYP 3A4 expressed in *Pichia pastoris* (Fessner et al., 2021). However, the limited number of potential redox partners for heterologous CYPs and imbalance of NADPH often requires co-expression of foreign CPRs for an effective enzyme function (Hausjell et al. 2018). Due to the low stability of CYPs, their susceptibility to the membrane environment and complex mechanism of the reaction, the productivity might depend on every single CYP to be expressed. In addition, microbial cells are cultivated under sterile conditions. This increases the process expenses as compared to the plants propagated on farm or in a greenhouse.

Plants provide an attractive assortment of prospective host systems for the expression of recombinant CYPs. In plants, several human CYPs were expressed with their native signal-anchor sequences and shown to be active without co-expression of cognate reducing partners (see, for example, (Sheludko et al. 2018, 2020; Azab et al. 2018; Warzecha et al. 2010, 2007). The introduction of CYPs with broad substrate specificity into plant metabolic pathways can lead to the diversification of natural product spectra (Frabel et al. 2018; Dueckershoff et al. 2005). Plants expressing human CYPs acquire resistance to herbicides (Anwar et al. 2011; Kawahigashi et al. 2008) and may serve as tools for phytoremediation (Azab et al. 2016, 2018). Recently, transient or stable plant expression systems were utilized for bioconversion of exogenous substrates by human CYPs into new compounds (Sheludko et al. 2018, 2020) or derivatives with improved pharmacological activities (Hidalgo et al. 2017; Martinez-Marquez et al. 2016). CYPs are capable of both detoxification and activation of natural products (Rendic and Guengerich 2021). Thus, individual human CYPs heterologously expressed in plants may be utilized for the precise conversion of pro-drugs into physiologically active compounds. For several human CYPs, the function and substrate scope is not conclusively defined yet. Their expression in plants may help to reveal possible substrates and to assess the pathways of drug metabolism.

Expression of CYPs in plants may offer an advantage of direct access to the energy generated by photosynthesis. CYPs can be relocated to the thylakoid membranes and coupled to the photosynthetic electron transport chains (Lassen et al. 2014) though the effect of chloroplast targeting is not obligatory advantageous to the CYP activity (Sheludko et al. 2018; Gnanasekaran et al. 2015). Productivity of the heterologous CYPs can be hampered by competition from primary pathways and inefficient electron transfer (Mellor et al. 2017, 2019). Recent reports also indicate a strong dependence of the activity of heterologous CYPs in chloroplasts on the molecular structure of the signal-anchor transmembrane domain and chloroplast transit peptide (de Jesus et al. 2017; Gnanasekaran et al. 2015). Modification of the microsomal leader domain enabled CYP catalytic function in chloroplasts but strongly reduced the transport capacity (Gnanasekaran et al. 2015). In our experiments employing transient expression in *Nicotiana benthamiana*, the conversion of a substrate by CYP2D6 and CYP3A4 bearing the native signal-anchor sequences was considerably higher in the case of ER localization as compared to the chloroplast targeting (Sheludko et al. 2018). Based on these results, we investigated two possible approaches to improve plant-based expression systems for human CYPs.

On the one hand, we established stably transformed hairy roots expressing in the nucleus the gene of CYP2D6 with native signal-anchor sequence. Transgenic hairy roots, induced after genetic transformation of plant cells with *Agrobacterium rhizogenes*, can be regarded as a promising alternative host for the expression of heterologous *cyp* genes (Hidalgo et al. 2017; Banerjee et al. 2002). As compared to the cultures of undifferentiated cells, hairy roots are genetically and metabolically stable and can actively grow on a phytohormone-free medium (Häkkinen et al. 2016).

As the second approach, the *cyp2D6* gene was integrated into the plastome to facilitate the compartmentalization of this protein in chloroplasts. Plastome transformation is known to provide a consistently high level of transgene expression due to precise integration of the transgene and the absence of gene silencing (Jensen and Scharff 2019; Zhang et al. 2017). Multiple recombinant proteins of industrial or pharmacological value were produced in plant chloroplasts (Daniell et al. 2021), but, to the best of our knowledge, a single report exists on the introduction of CYP genes (CYP79A1, CYP71E1 from *Sorghum bicolor*) into the plastome of *Nicotiana tabacum* engineered for the biosynthesis of cyanogenic glucoside dhurrin (Gnanasekaran et al. 2016). Here we report for the first time the integration of a human CYP with broad substrate specificity into chloroplast genome and regeneration of transplastomic plants expressing physiologically active CYP2D6. The efficiency of CYP2D6 expression in hairy root cultures and transplastomic plants was estimated at the transcriptional and physiological level and compared with the data of CYP2D6 transient expression in *N. benthamiana*.

## Materials and methods

### Genetic constructs

The protein sequence, annotated as CYP2D6 (NCBI Ref. No. XP_016885819) in the reference sequence human genome assembly, was back-translated with codon optimization for expression in *N. tabacum* using the OptimumGeneTM (GenScript, USA) algorithm, and the nucleotide sequence was synthesized by GenScript. The bifunctional reporter gene *GFP::licBM3* (NCBI Ref. No. KX458181) was created by a fusion of the sequences encoding GFP and the truncated thermostable lichenase from *Clostridium thermocellum* (Gerasymenko et al. 2019).

Genetic vectors for nuclear transformation and transient expression were assembled using the Golden Braid modular cloning system (Sarrion-Perdigones et al. 2013). For transient expression, the plasmids of omega level included transcriptional units for the genes of interest and the p19 suppressor of post-transcriptional gene silencing of tomato bushy stunt virus under the control of 35S CaMV promoter and nopaline synthase gene terminator from *A. tumefaciens* (Fig. S3.1a,b (Sheludko et al. 2018)).

For stable nuclear transformation and establishment of *N. tabacum* hairy root cultures, the transcriptional units of the *p19* gene were exchanged for the transcriptional units of the selective neomycin phosphotransferase II (*npt*II) gene under the control of the nopaline synthase gene promoter and 35S CaMV terminator (Fig. S3.1c).

**Fig. 1:**
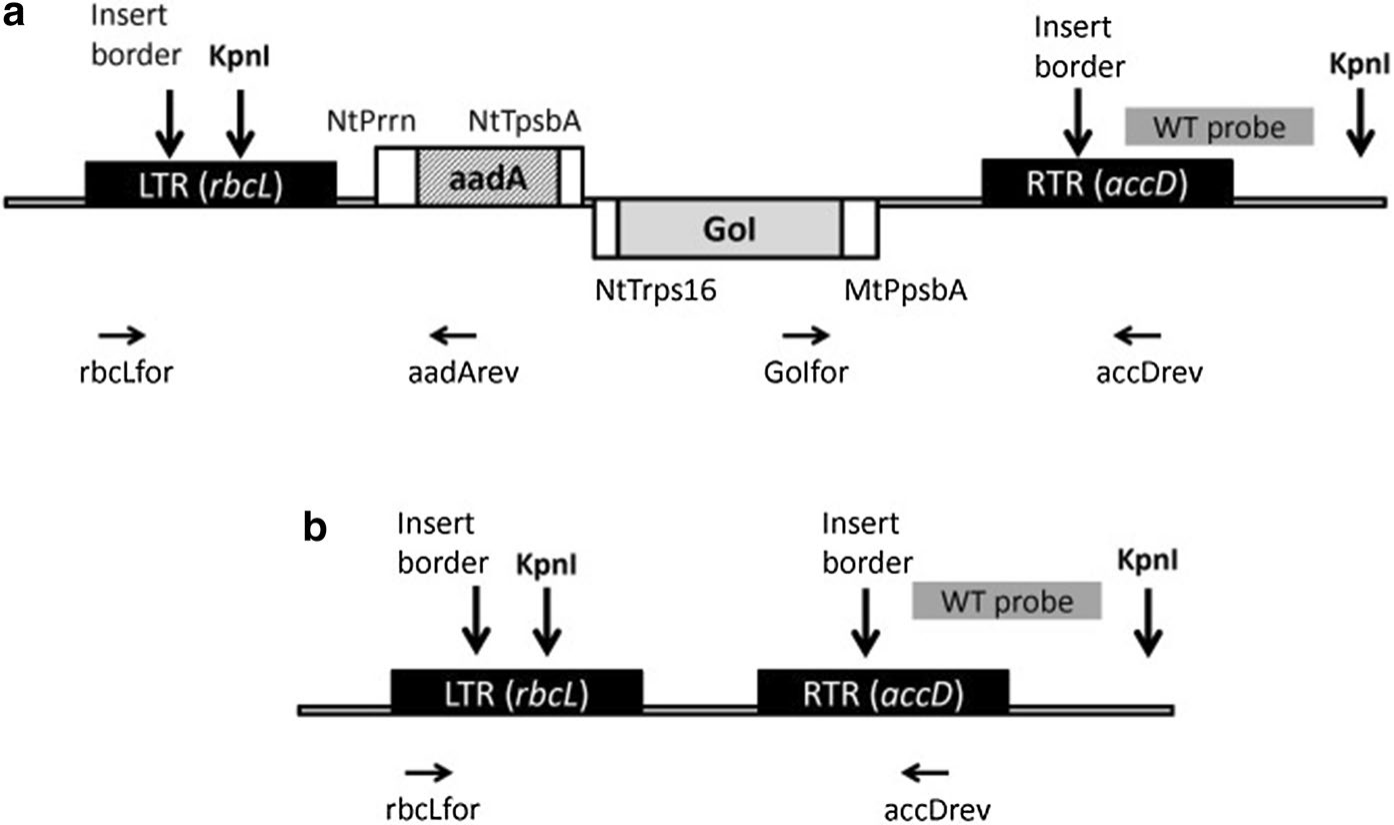
Fragments of *N. tabacum* plastome with the insert (**a**) and of the wild-type (**b**). Horizontal arrows indicate the primers flanking the insert (rbcLfor and accDrev) and the transgene-specific primers (aadArev and GFPfor/2D6for) applied for PCR analysis. Vertical arrows show the insert borders and the KpnI cutting sites; a wild-type (WT) probe was used to visualize RFLP by Southern blot hybridization. LTR, left targeting region; RTR, right targeting region; NtPrrn, *rrn* promoter of *N. tabacum*; MtPpsbA, *psbA* promoter and 5′-UTR of *Medicago truncatula*; NtTpsbA, *psbA* 3′-UTR of *N. tabacum*; NtTrps16, *rps16* 3′-UTR of *N. tabacum*; aadA, aminoglycoside 3′-adenyltransferase coding sequence; GoI, gene of interest (*GFP::licBM3* or *cyp2D6*)

The constructs for plastome transformation were assembled using a modified Golden Braid toolbox (Vafaee et al. 2014). The alpha level plasmids contained the transcriptional units for the genes of interest driven by 5′-UTR of the *psbA* gene of *Medicago truncatula* and the 3′-UTR of *rps16* gene of *N. tabacum* (Gerasymenko et al., 2017) and for the *aadA* gene controlled by *rrn* promoter and *psbA* 3′-UTR of *N. tabacum*. The transcriptional units were flanked by the parts of *rbcL* and *accD* operons of the tobacco plastome for homologous recombination (Fig. 1).

Assembly of the genetic constructs was performed in *E. coli* DH5α strain.

### Genetic transformation and transient expression

Transient expression of CYP2D6 was carried out in leaves of *N. benthamiana* using *A. tumefaciens* GV3101 strain as described earlier (Gerasymenko et al. 2019).

Biolistic transformation of *N. tabacum* in vitro leaves was carried out with a Biolistic® PDS-1000/He Particle Delivery System (Bio-Rad, USA) using 1100 psi rupture discs. Plasmid DNA (10 µg in 10 µl) was applied onto the gold microparticles (0.6 μm) via spermidine and CaCl_2_ precipitation. After bombardment, the leaves were incubated for two days on the solidified RMOP medium without antibiotics and thereafter cut into small pieces. The explants were placed onto the RMOP medium containing spectinomycin and streptomycin (300 mg/l each) and maintained under light at 25 °C. The emerging green calli were detached, transferred onto RMOP containing spectinomycin and streptomycin (500 mg/l each). For plant regeneration, the calli were cultivated on the solidified MS medium containing 500 mg/l spectinomycin. The formed plants were transferred into the greenhouse and maintained at 23 ± 2 °C and 60% humidity with the 16/8 h light/dark period. The seeds were obtained by self-pollination, sterilized with 6% sodium hypochlorite and germinated on the solidified MS medium containing spectinomycin and streptomycin (500 mg/l each). After four weeks, the green plantlets were transferred onto the solidified MS medium containing 500 mg/l spectinomycin. After a month, the T1 plants were transferred into the greenhouse and maintained as described above during four weeks before analysis.

For induction of hairy roots, the leaf explants of in vitro plants *N. tabacum* cv. Petit Havana were infected with *A. rhizogenes* A4 strain (see Supplementary materials S1.1 for details).

### DNA analysis

Total DNA was isolated from plant material (200 mg leaf tissue or 500 mg roots) using CTAB (Querci et al. 2020). For PCR analysis, 100 ng of total DNA were applied. The amplification was carried out with DreamTaq DNA polymerase in the corresponding buffer (ThermoScientific). The reaction mixture in a total volume of 20 µl contained 0.5 mM dNTPs (0.125 mM each), 0.25 µM of each primer (Table S2.1), and 0.5 units DreamTaq DNA polymerase. For detection of rolB/C and virD1 genes, the reaction was carried out under the following conditions: initial denaturation at 95 °C for 5 min → 30 cycles including denaturation at 95 °C for 30 s, annealing at 56 °C for 30 s, elongation at 72 °C for 60 s) → final elongation at 72 °C for 5 min. For detection of *GFP::LicBM3* and *CYP2D6* genes in the hairy root cultures, the annealing temperature was set at 65 °C, and the elongation time was extended to 90 s. For analysis of plastome DNA, the reaction was carried out with the annealing temperature of 65 °C and the elongation time of 3 min.

For restriction fragment length polymorphism analysis by Southern blot hybridization, the total DNA (500 ng) was digested with KpnI enzyme and separated in the 1% agarose gel using 1 × TAE buffer. The apurinated DNA was transferred onto the Roti®-Nylon plus membrane (Roth, Germany) by capillary transfer in 20 × SSC buffer, cross-linked and hybridized at 42 °C in DIG Easy Hyb solution (reconstituted from DIG Easy Hyb granules, Roche, Switzerland) with digoxigenin-labelled probes. The probes were prepared using PCR DIG probe synthesis mix (Roche, Switzerland) and primers ptNtWT1 5′-cattgaatttcattcggaggaggag-3′ and ptNtWT2 5′-ATCGAAAGATTCGTTCGAATCCGAC-3′. Chemiluminescent detection was carried out using anti-digoxigenin-AP FAB fragments and CDP Star ready-to-use solution (both from Roche, Switzerland).

### Quantitative reverse transcription-PCR

RNA was isolated from 100 mg of plant material using 1 ml of peqGOLD TriFast (VWR). For cDNA synthesis, 2 µg of total RNA were applied. The RNA samples were treated with TURBO DNase (Invitrogen, Thermo Fisher Scientific), mixed with an oligoT (5′-T15-NNN-3′) or a random hexamer primer (for transplastomic plant samples), heated at 65 °C for 5 min, and put on ice. To the reaction mixture in a total volume of 20 µl were added dNTPs (0.5 mM, 0.125 mM each), SuperScript IV reverse transcriptase (200 units, Invitrogen, Thermo Fisher Scientific) with the corresponding buffer, and RiboLock RNase inhibitor (20 units, Invitrogen, Thermo Fisher Scientific). The first strand cDNA synthesis was carried out at 55 °C for 30 min; the enzymes were inactivated at 80 °C for 10 min. For qPCR analysis, the cDNA preparations were diluted at 1:100 with deionized water. The reaction mixture in a total volume of 20 µl contained 10 µl of PowerUp SYBR Green Master Mix (Applied Biosystems, Thermo Fisher Scientific), 1.25 µM of each primer (the sequences see below), and 2 µl of a template cDNA. The reaction was carried out in a StepOne thermocycler (Applied Biosystems, Thermo Fisher Scientific) under the following conditions: initial denaturation at 95 °C for 20 s; 40 cycles of 95 °C for 3 s and 60 °C for 30 s; melting curve recording by changing the temperature from 60 to 95 °C in 0.3 °C increments. The results were analyzed with StepOne Software v. 2.3 (Applied Biosystems, Thermo Fisher Scientific). The reaction for each sample was run in two technical repeats. For hairy roots, three samples were analyzed for each line. For each transplastomic line, samples from five individual plants were prepared. The transient expression experiments were performed in four reiterations.

The efficiency of each primer pair was determined using 10-fold serial dilutions of cDNA samples. The threshold cycle (Ct) values of each dilution were plotted against the logarithm of the dilution factor. The amplification efficiency was calculated by the formula:

$$\% {\text{E}} = \left( {10^{{ - 1/{\text{slope}}}} - \, 1} \right) \times 100\%$$

The percentage efficiencies of reference gene primer pairs retrieved from literature were 109.3% (nuclear NtEF1α; Schmidt and Delaney 2010) and 108.8% (plastome NtIN1; Cortleven et al. 2009). The primer pairs designed for amplification of transgene fragments showed efficiencies of 106.4% (LicBM3; Fw 5′-agctgccaaaaacgtagg-3′, Rv 5′- TGGATTGTTGTCCGAAGG-3′) and 99.1% (cyp2D6; Fw 5′- ctgagcataggatgacatgg-3′, Rv 5′- CCTCAGTCAAATCTCTAGGTG-3′).

The relative transcript levels were calculated by raising 2 in power of ΔCt (Ct value of reference gene—Ct value of transgene).

### Western blot analysis

For Western blot analysis, the explants of approximately 100 mg were cut from the leaves using a cylindrical metal punch and ground in 500 μl of 2-fold SDS-PAGE loading dye. After 10 min heat denaturation at 95 °C, the samples were separated in a 12% polyacrylamide gel and transferred onto a Roti-PVDF membrane with pore size 0.45 µm (Roth, Germany). The hybridization was carried out with anti-GFP mouse monoclonal primary antibody and goat anti-mouse secondary antibody fused with horseradish peroxidase (both from Santa Cruz Biotechnology; 1:10,000 dilution). For detection, the CheLuminate-HRP PicoDetect kit (AppliChem, Germany) was applied.

### Lichenase activity assay

Analyses of GFP::LicBM3 activity was carried out as described earlier (Gerasymenko et al. 2019).

### Feeding experiments

For routine loratadine (LOR) biotransformation experiments, we used commercially available tablets (Lora-ADGC1, KSK-Pharma AG, Berghausen, Germany).

Eight transplastomic plants, grown in a greenhouse (four carrying GFP::licBM3 and four carrying *cyp2D6* gene), each derived from an individual line of green callus, were investigated. For infiltration, LOR tablets were extracted as described (Sheludko et al. 2018), and 50 μl of tablet extract was mixed with 950 μl of the infiltration buffer, containing 10 mM MES and 10 mM MgSO_4_ at pH 5.5. Leaves were infiltrated with LOR solution or infiltration buffer as a control, and the procedure was repeated after four days. The leaf material was collected after seven days. For the analysis of total leaf tissue, discs of 25 mm diameter (60–110 mg) were cut with a cylindrical metal punch, frozen in liquid nitrogen and lyophilized. The chloroplasts were isolated from approximately 1 g of leaf material as described (Kley et al. 2010), frozen in liquid nitrogen and lyophilized. Metabolites were extracted as described (Sheludko et al. 2018) with minor modifications (see Supplementary materials S1.2).

Hairy roots, maintained on the solidified MS medium without selective agents for 30–35 days, were inoculated (0.2–0.4 g) into the liquid medium B5 (Gamborg et al. 1968), supplied with a half concentration of inorganic salts and 15 g/l sucrose, and cultivated on a rotary shaker (40–50 rpm) at 25 °C and low light. On the 6th day, the culture medium was replaced with a fresh volume of 5 ml. LOR tablet was dissolved in 1 ml of 70% EtOH by vortexing for 60–90 s and then extracted for 15 min at room temperature and 750 rpm. After centrifugation for 5 min at 17,000 × g, the supernatant was added to the culture medium (50 µl per flask containing 5 ml of the culture medium), giving the final concentration of LOR 19 µg/ml (49 µM). Additional CYP2D6 substrate, indole alkaloid corynanthine ((Sheludko et al. 2018), Fig. S2), was added to the culture medium giving the final concentration of 20 µg/ml (57 µM).

Hairy root biomass and culture medium were harvested 7 days post substrate administration. Hairy roots were freeze-dried and ground to a fine powder. The biomass samples (10 mg) were twice extracted with 750 µL of MeOH for 60 min under sonication at 30–35 °C. Extracts were combined, centrifuged, and the supernatants were analysed by HPLC–MS.

The cultural medium (5 ml) was filtered, and pH was adjusted to the alkaline range (9.5–10) with 32% ammonium solution. The medium was extracted with an equal volume of ethyl acetate. After phase separation, the organic fraction was filtered through anhydrous sodium sulfite. The aliquot of 1 ml was evaporated, dissolved in 200 ml of MeOH and HPLC–MS-tested.

For calculation of the conversion rate, initial LOR concentration in leaves was determined as a molar sum of LOR and DCL in extracts on the base of HPLC data; initial LOR concentration in hairy root cultures was calculated from the supplemented volume of LOR tablet extract.

### Analytical assays

HPLC and HPLC–MS analyses of LOR metabolites were carried out as described earlier (Sheludko et al. 2018). To normalize the results for possible deviations of the substrate concentration in the leaf tissue, conversion rates were estimated as a molar percentage of total (substrate and products) amounts of LOR metabolites in the sample, calculated on the base of areas of corresponding peaks on HPLC chromatogram compared to standard curves built in the range of 1–75 ng.

Secondary metabolite profiles in extracts of transplastomic plants were analyzed by HPLC using the 1260 Infinity system connected to a Zorbax Eclipse XDB-C18 column, 4.6 × 250 mm, 5 μm (Agilent). The mobile phase consisted of 0.1% (v/v) formic acid with 10 mM hexane sulfonic acid (A) and acetonitrile (B). Separation was carried out applying the following binary gradient elution program (% B): 5 within 5 min; 5–100 within 20 min. The column was flushed with 100% B for 5 min and re-equilibrated with 5% B for 5 min; the flow rate was set to 1.0 ml/min. To detect substances in plant extracts, UV absorption at λ = 260 nm was used.

### Quantification of chlorophyll and carotenoid pigments

For the determination of photosynthetic pigment content, leaf samples (100 mg) were extracted with 1 ml of acetone. For each transplastomic line, four individual plants were analysed. The concentrations of chlorophylls a and b and total carotenoids were calculated based on absorption values at 661.6, 644.8 and 470 nm using formulas from Lichtenthaler (Lichtenthaler 1987).

### Statistical analysis

The data are represented as an average mean ± standard deviation. Standard deviations were calculated using Microsoft® Office Excel 2010 standard functions. Statistical significance of average mean divergences was estimated with Student’s two-sample *t*-test (two-tailed distribution; unequal variance); *p* values were calculated using Excel standard functions. Sample sizes are given in the description of each experiment and are mentioned in the figure and table captions. The maximal probability of the data compatibility with the null hypothesis α  = 0.05 is defined as the significance level for significant finding. In the text, we indicated also the experiments where we registered a significance value exceeding the next commonly used threshold level (α  = 0.01).

## Results

### Generation of transplastomic lines and confirmation of homoplasmy

Genetic constructs for plastome transformation were assembled using the Golden Braid cloning system (Vafaee et al. 2014). To create a physiologically neutral control, plants were transformed with a gene encoding the bifunctional reporter protein consisting of GFP and thermostable lichenase (GFP::LicBM3) (Gerasymenko et al. 2019). Expression of genes of interest was controlled by 5′-UTR of *pbsA* gene of *Medicago truncatula* and 3′-UTR of *rps16* gene of *N. tabacum* (Gerasymenko et al. 2017); the *aadA* gene was driven by *rrn* promoter and *psbA* 3′-UTR of *N. tabacum*. The transcriptional units were inserted between *rbcL* and *accD* operons of tobacco plastome (Fig. 1).

The green calli formed after biolistic transformation on RMOP medium containing spectinomycin and streptomycin were maintained on the same medium. After 9 months, the DNA isolated from green calli was analyzed by PCR for detection of the *aadA* gene and the transgenes of interest. A combination of gene-specific primers and primers flanking the integration site was used to prove the integration of the insert into the expected site. Amplification with the flanking primers was carried out to reveal the presence of wild type plastomes (Fig. 1).

**Fig. 2:**
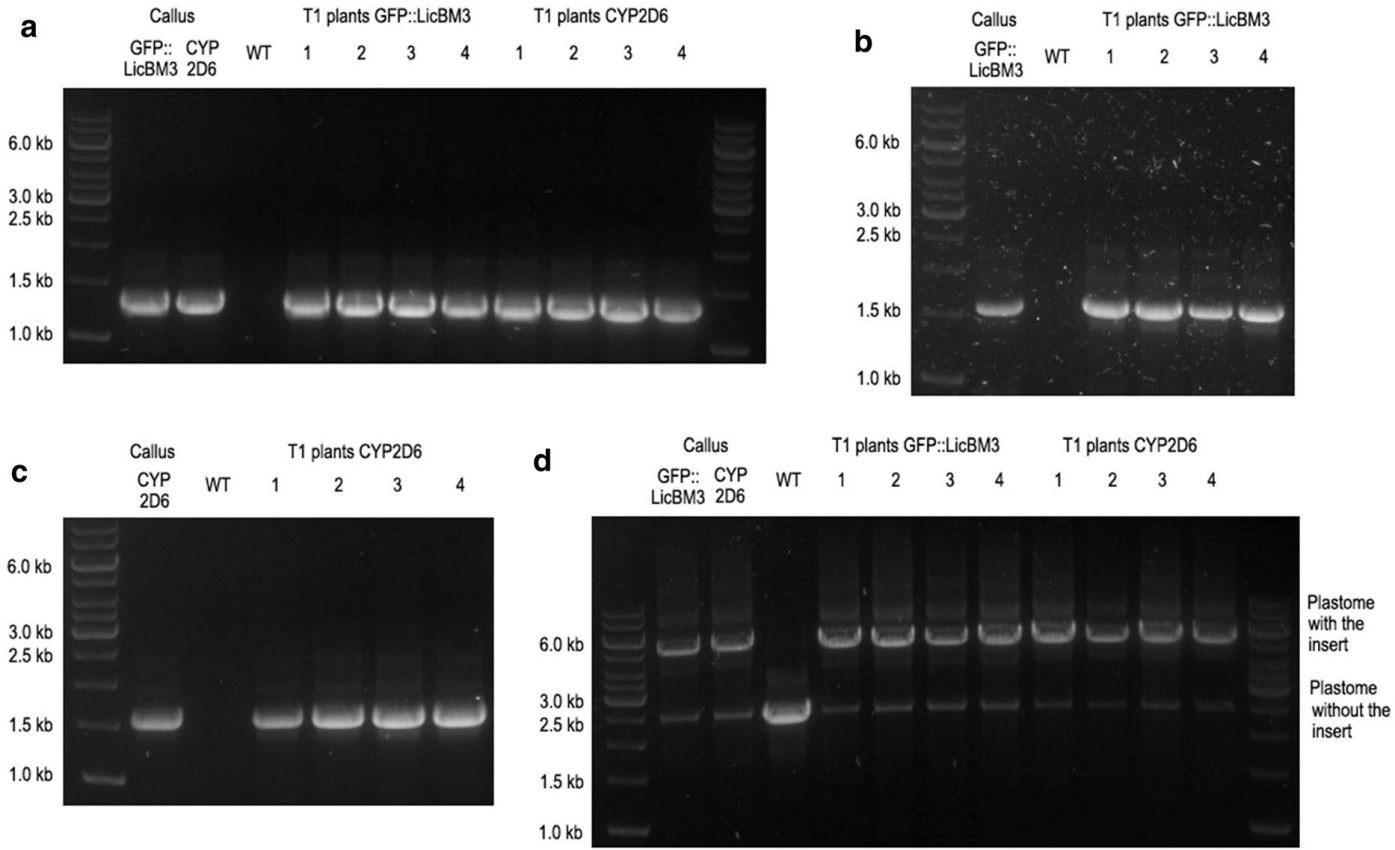
PCR analysis of *N. tabacum* transplastomic callus lines carrying *GFP::licBM3* and *cyp2D6* genes and T1 plants (four T1 plants derived from one callus line). WT: DNA from wild-type *N. tabacum* leaves. **a** Reaction with a primer specific for the *aadA* gene (aadArev) and a primer flanking the insert site (rbcLfor), proving the site-specific integration of the *aadA* gene. **b** Reaction with a primer specific for the *GFP::licBM3* gene (GFPfor) and a primer flanking the insert site (accDrev), proving the site-specific integration of *GFP::licBM3* gene. **c** Reaction with a primer specific for the *cyp2D6* gene (2D6for) and a primer flanking the insert site (accDrev) proving the site-specific integration of the *cyp2D6* gene. **d** Reaction with primers flanking the insert site (rbcLfor and accDrev) revealing the transgenic plastomes with the insert and the wild-type plastomes without the insert

For all selected green calli (six lines carrying the reporter gene and eleven lines with the *cyp2D6* gene), the correct integration of the *aadA* selection marker and the genes of interest was confirmed; however, a fragment indicating the residual presence of the wild type plastomes was also amplified (examples are shown in Fig. 2). After 18 months, phenotypically normal plants were regenerated on MS medium with spectinomycin, rooted on MS medium without antibiotics and transferred into the greenhouse. The seeds formed after self-pollination were collected and germinated on MS medium containing spectinomycin and streptomycin. After three weeks, only green plantlets were observed (Fig. S3.2). The plantlets (T1 generation) were placed onto MS medium with spectinomycin, and a month later four transplastomic plants of each line were transferred into the greenhouse. Transplastomic plants expressing CYP2D6 were developing slower under greenhouse conditions as compared to the control group transformed with a physiologically neutral reporter gene, contained significantly (α = 0.05) less chlorophylls a and b (Fig. S3.3) but were able to form normal flowers and fertile seeds.

After a month of cultivation in soil, the PCR analysis was repeated with the DNA isolated from leaves. For all analysed T1 plants, the absolute predominance of the transplastomic chloroplasts over the wild type plastome was confirmed (Fig. 2).

**Fig. 3:**
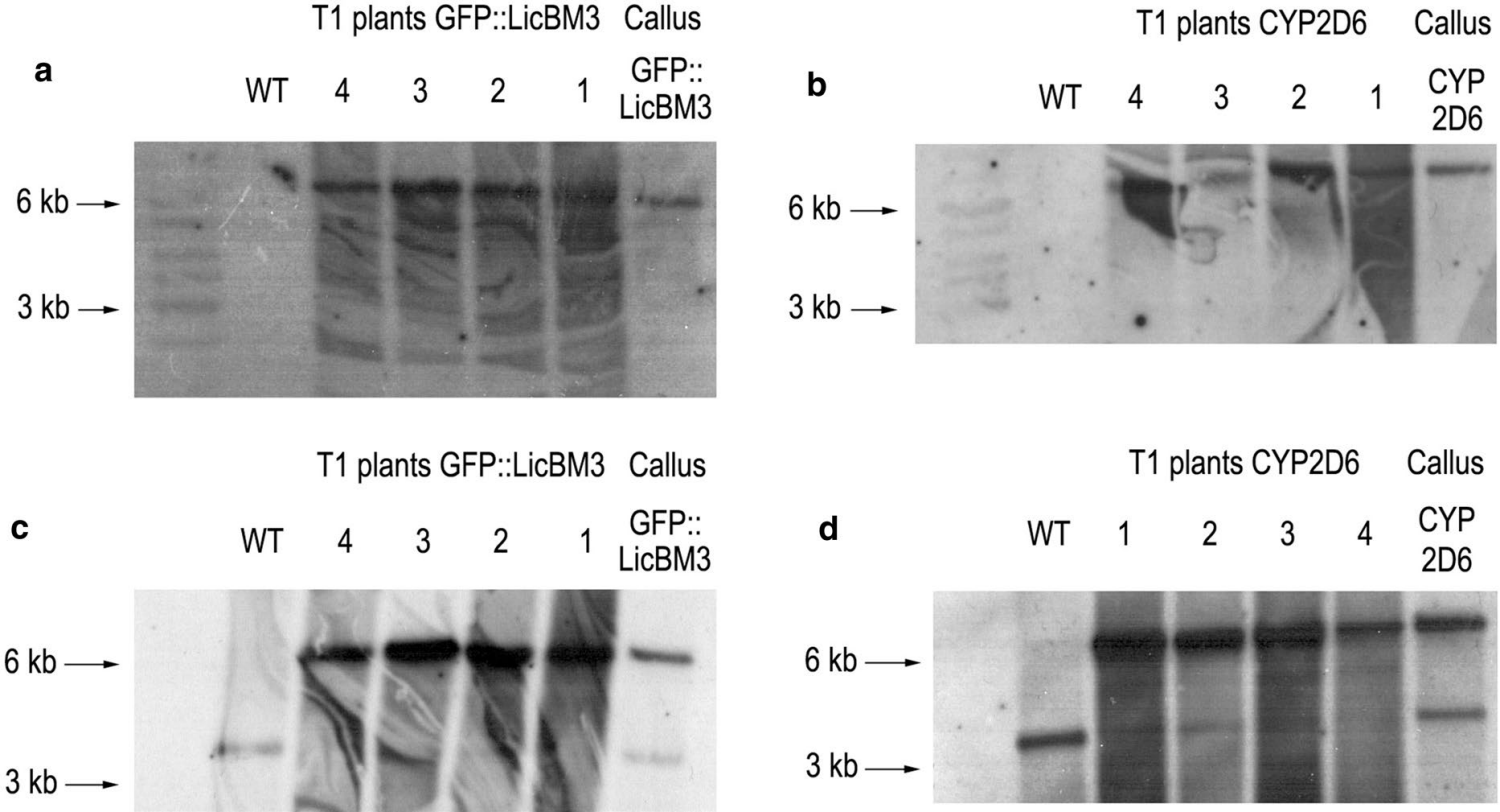
Southern blot analysis of *N. tabacum* transplastomic callus lines carrying *GFP::licBM3* and *cyp2D6* genes and T1 plants (four T1 plants derived from one callus line). WT: DNA from wild-type *N. tabacum* leaves. **a** Hybridization with a probe complementary to the *GFP::licBM3* gene. A single band of 6.2 kb after digestion of total DNA (500 ng) with KpnI confirms unique site-specific integration of the insert. **b** Hybridization with a probe complementary to the *cyp2D6* gene. A single band of 6.4 kb after digestion of total DNA (500 ng) with KpnI confirms unique site-specific integration of the insert. **c**, **d** Hybridization with a probe complementary to the *accD* operon of *N. tabacum* plastome (WT probe). After digestion of total DNA (500 ng) with KpnI, a band of 3.5 kb derives from the wild type plastome; the bands of 6.2 and 6.4 kb corresponding to the plastomes carrying the *GFP::licBM3* and *cyp2D6* genes, respectively

The DNA isolated from initial calli and T1 plants (after a month of cultivation in the greenhouse without selection) was assessed for RFLP by Southern blot hybridization with a transgene specific probe to prove that the insert integrated only in the expected site (Fig. 3a, b). Hybridization with a probe complementary to the *accD* operon displayed a factually homoplasmic state of T1 plants (Fig. 3c, d).

### Generation and analysis of hairy root cultures bearing the gene of CYP2D6

**Fig. 4:**
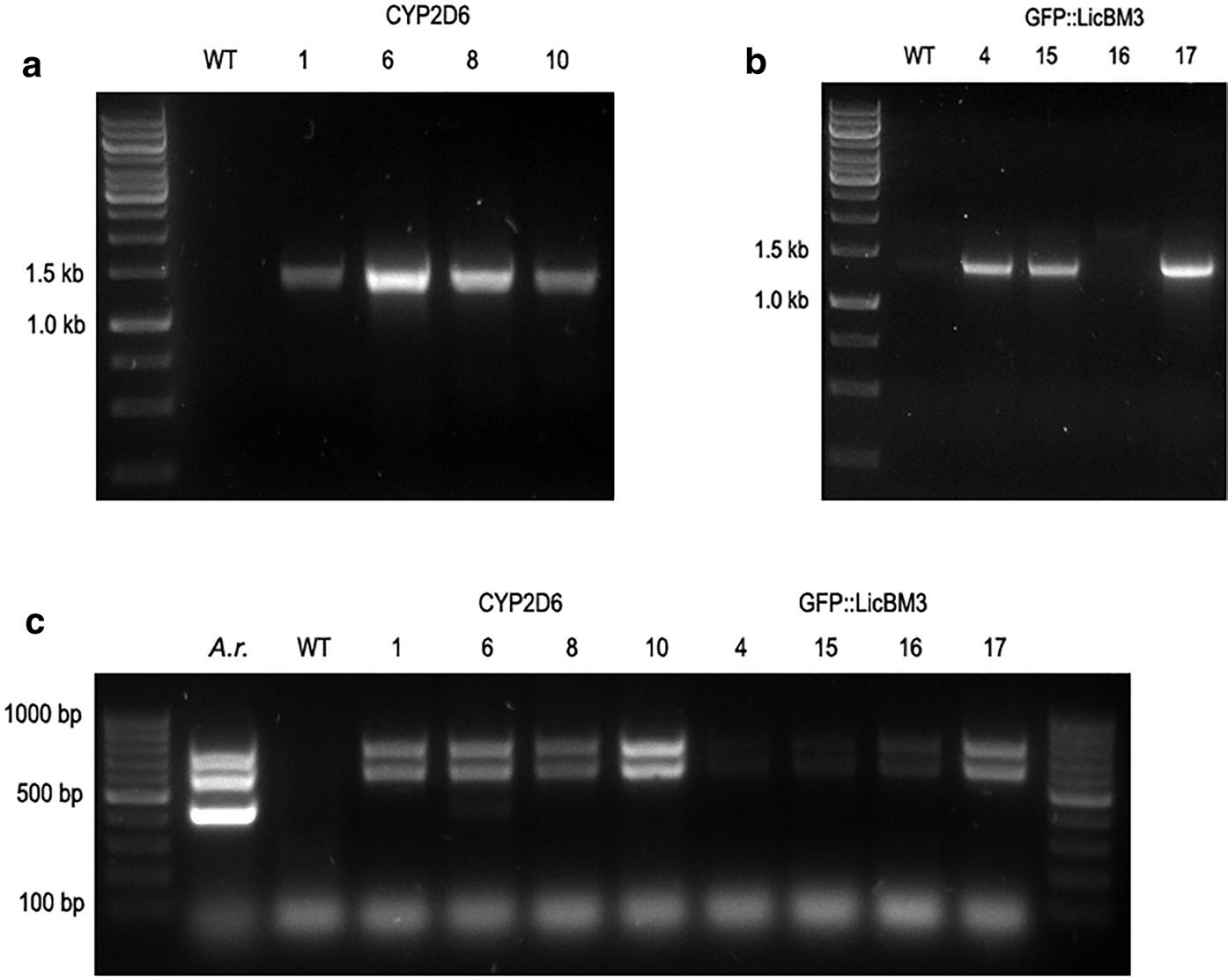
PCR detection of genes involved in hairy root formation (*rolB* and *rolC*) and genes of interest (*cyp2D6* and *GFP::LicBM3*) in *N. tabacum* hairy root cultures; absence of *virD1* gene proves elimination of *A. rhizogenes*. **A** Fragment of 1529 bp amplified after the reaction with primers specific for the *cyp2D6* gene (2D6for and 2D6rev); **B** fragment of 1406 bp amplified after the reaction with primers specific for the *GFP::licBM3* gene (GFPfor and GFPrev); **C** fragments of 432 bp, 586 bp and 776 bp amplified after the reaction with primers specific for the *vir*D1 gene (virDfor and virDrev), *rol*C gene (rolCfor and rolCrev) and *rol*B gene (rolBfor and rolBrev), respectively

Hairy root cultures of *N. tabacum* were obtained by transformation with *A. rhizogenes* A4 strain carrying the genetic constructs with either reporter *GFP::licBM3* or *cyp2D6* gene bearing the native signal-anchor sequence. After selection, roots growing on hormone-free solidified MS medium were subjected to PCR analysis that proved the presence of genes of interest as well as *rolB* and *rolC* genes involved in the formation of hairy root phenotype (Fig. 4). Three lines carrying the reporter *GFP::licBM3* gene and four lines with the *cyp2D6* gene were selected for further experiments.

### Transcription rate of the transgenes in transgenic and transplastomic plants and transient expression system

**Fig. 5:**
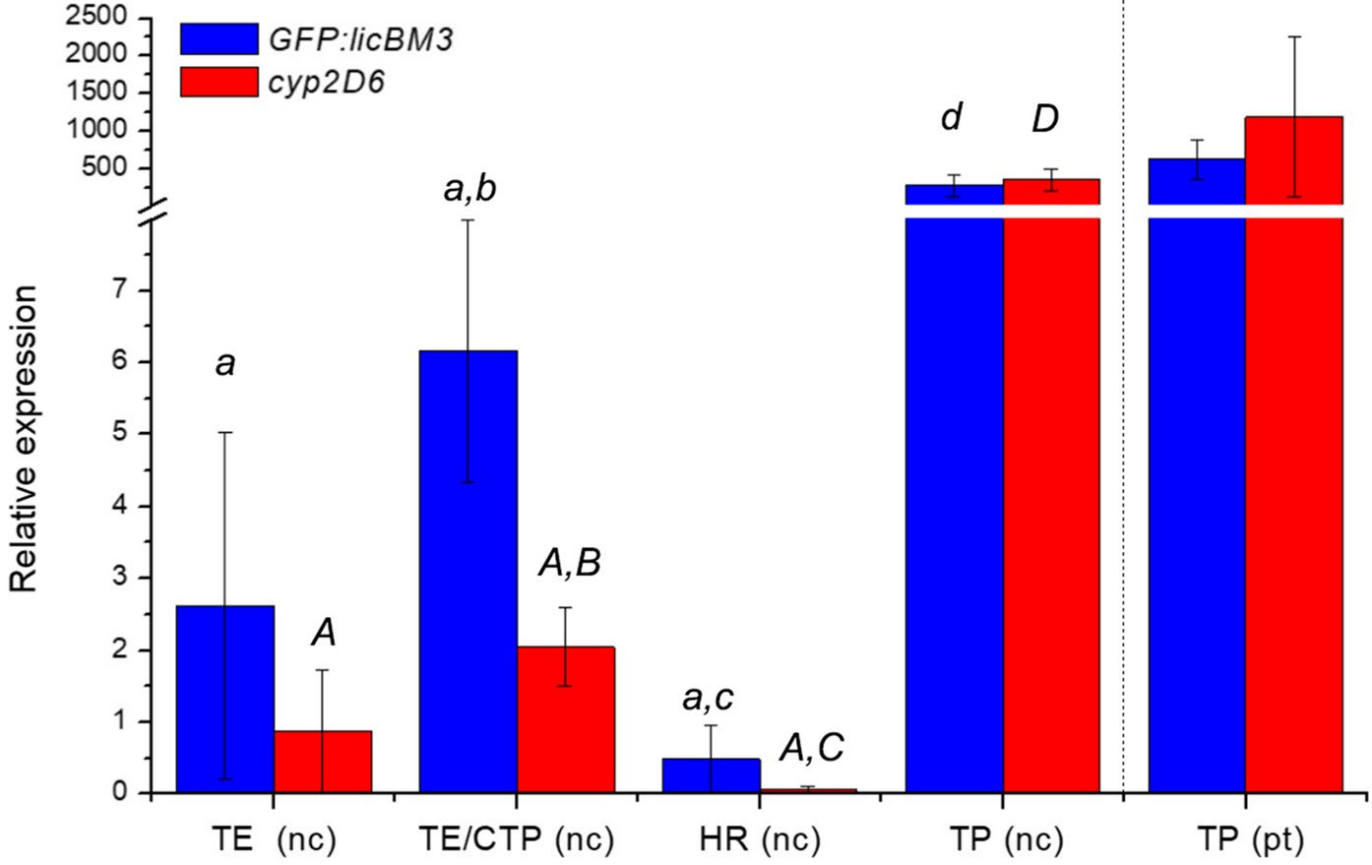
Relative transgene transcript levels normalized to the nuclear *NtEF1α* (nc) and the plastomic *NtIN1* (pt) gene. TE: Leaves of *N. benthamiana* transiently expressing the transgenes without a sequence encoding chloroplast targeting peptide (mean of four biological repeats for each gene); TE/CTP: leaves of *N. benthamiana* transiently expressing the transgenes with a sequence encoding chloroplast targeting peptide (mean of four biological repeats for each gene); HR: transgenic hairy root cultures of *N. tabacum* (mean of four transgenic lines (*cyp2D6*) or three transgenic lines (*GFP::LicBM3*), three biological repeats of each); TP: transplastomic T1 plants of *N. tabacum* (mean of five plants). Bars represent standard deviations. Significances of difference between *GFP::LicBM3* and *cyp2D6* expressing groups are not shown. The difference between transcript levels in transplastomic plants normalized to the nuclear *NtEF1α* (nc) and the plastomic *NtIN1* (pt) genes is not significant. Different letters indicate significant differences at α = 0.05 (lowercase) and α = 0.01 (capital). For details, refer to the Table S2.2

Real-time quantitative PCR (RT-qPCR) was employed to analyse the expression of transgenes, *GFP::licBM3* and *cyp2D6*, on the transcriptional level in the leaves of transplastomic T1 *N. tabacum* plants, transgenic hairy root cultures and in the leaves of *N. benthamiana* transiently expressing the reporter and *cyp2D6* genes with native microsomal leader sequence or with N-terminally fused CTP sequence. For normalization of RT-qPCR data, we used reference gene reported to be stably expressed in nucleus (*NtEF1α*; (Schmidt and Delaney 2010)). For transplastomic plants we additionally employed as a reference *NtIN1* gene (Cortleven et al. 2009) expressed in the plastom of *N. tabacum*, but the difference between transcript levels normalized to the nuclear or plastomic reference genes was not significant (Fig. 5).

(*p* ≤ 0.016). The relative transcript levels of the reporter *GFP::licBM3* and *cyp2D6* genes in the leaves of T1 transplastomic plants surpassed manifold the levels observed after constitutive or transient expression from the nucleus, with or without a sequence encoding CTP (Fig. 5). The relative levels of transgene transcripts varied in transgenic hairy root lines (Fig. S3.4), but in all of them the values were significantly lower than in transiently transformed leaves expressing the transgenes with the CTP encoding sequence or in transplastomic plants (Fig. 5, see also values in Table S2.2).

### Functional activity of transgenes in transplastomic plants and hairy root cultures of *N. tabacum*

#### Biotransformation of loratadine in transplastomic plants of *N. tabacum*

**Fig. 6:**
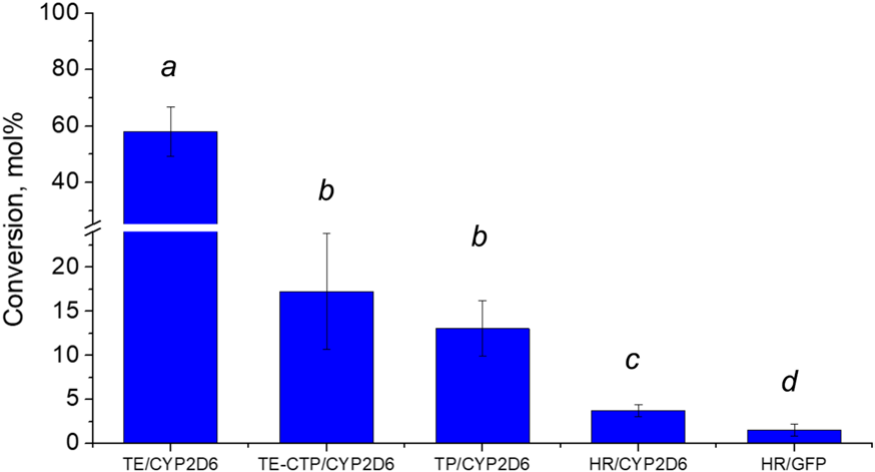
Conversion of LOR in transplastomic plants (TP/CYP2D6, plants transformed with *cyp2D6*, mean of eight plants) and hairy root cultures (HR/CYP2D6, hairy root cultures transformed with *cyp2D6*, mean of four transgenic lines; HR/GFP, hairy root cultures transformed with *GFP::licBM3*, mean of three transgenic lines) of *N. tabacum* in comparison with transient expression systems (*N. benthamiana* plants transiently expressing transgene with native leader sequence (TE/CYP2D6, mean of eight repeats) or fused to chloroplast targeting peptide sequence (TE-CTP/CYP2D6, mean of five repeats); the values for transient expression system retrieved from (Sheludko et al. 2018)). Bars represent standard deviations; different letters indicate significant differences at α = 0.05 (between TE/CYP2D6 and TE-CTP/CYP2D6) and α = 0.01 (for the rest)

Enzymatic activity of CYP2D6 in the studied plant expression systems was determined by its ability to convert a synthetic antihistaminic drug loratadine (LOR), a reported CYP2D6 substrate (Ghosal et al. 2009). CYP2D6 transforms LOR to descarboethoxyloratadine (DCL) (Fig. S3.5A). This reaction was studied in leaves of *N. benthamiana* transiently expressing CYP2D6. The highest DCL concentration was achieved when the LOR was administered two times, on the 1st and 4th day post *Agrobacterium* infiltration, and the plant material was collected on the 7th day post infiltration (Sheludko et al. 2018). In transplastomic plants, we used the same substrate infiltration scheme. Chromatographic analysis of the leaf extracts revealed a peak with the retention time and spectral characteristics identical to DCL only in the plant samples carrying the *cyp2D6* gene (Fig. S3.6). The LOR conversion rate was determined to be 13.04 ± 3.16 mol%, which is considerably lower than it was reported for transiently expressed CYP2D6 with the native ER signal-anchor sequence (58.06 ± 8.83 mol%), but is similar to that for the chloroplast targeted protein (17.26 ± 6.61 mol%) (Sheludko et al. 2018)) (Fig. 6). To prove that the observed low conversion rate is not due to the reduced availability of the externally supplied substrate within the chloroplasts, the chloroplasts of the infiltrated plants were isolated and extracted. LOR was recovered from the chloroplast extracts that proves its import into the organelle. However, only trace amounts of DCL were detected in the chloroplast extracts, probably due to the export of DCL into the cytoplasm (Fig. S3.7).

#### Biotransformation of loratadine by transgenic hairy roots of *N. tabacum*

For estimation of CYP2D6 catalytic activity, hairy roots expressing CYP2D6 and control lines transformed with GFP::licBM3 were cultivated in the liquid medium containing LOR and corynanthine. After seven days of incubation, the root biomass and the nutritious medium were analysed for the content of LOR, DCL and hydroxycorynanthine. The latter product was not detected in the extracts of hairy root cultures. Unlike transplastomic plants and transiently transformed leaves, DCL was identified in the extracts of tissue and culture medium of the control cultures, although the yield of DCL in extracts of cultures carrying *cyp2D6* gene surpassed significantly (α = 0.01) that in control (3.73 ± 0.65 mol% and 1.53 ± 0.71 mol%, respectively; Fig. 6). Subtracting background activity gives CYP2D6-specific yield of DCL in hairy roots as 2.2%. This value is almost one-fifth of the conversion rate in transplastomic plants and 25-fold lower than the value of the transient expression.

The substrate LOR and its conversion product DCL were differently distributed between the root tissue and the culture medium (Fig. S7). While the most part of LOR (> 95%) was extracted from the root material, suggesting an efficient import of exogenous LOR into hairy root cells, almost a quarter of the extracted DCL was found in the culture medium. (19.14 ± 6.49% and 24.34 ± 2.28% for *GFP::licBM3* and *cyp2D6* lines, respectively, Fig. S3.8).

### Analysis of lichenase activity

**Fig. 7:**
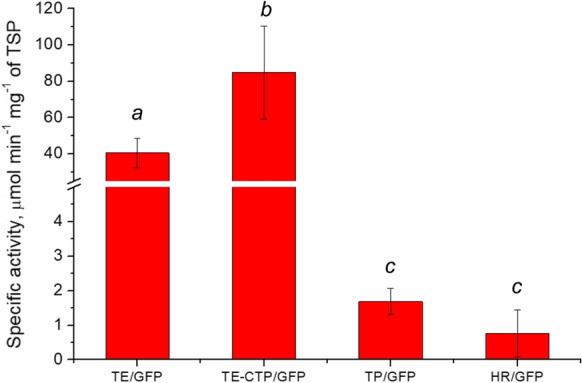
Functional activity of the reporter protein GFP::LicBM3 in transplastomic plants (TP/GFP, plants transformed with *GFP::LicBM3*, mean of four plants) and hairy root cultures (HR/GFP, hairy root cultures transformed with *GFP::LicBM3*; mean of three lines, each was measured in three repeats) of *N. tabacum* in comparison with transient expression systems (*N. benthamiana* plants transiently expressing the transgene without (TE/GFP, mean of eight repeats) or with chloroplast targeting peptide sequence (TE-CTP/GFP, mean of ten repeats); the values for transient expression system retrieved from (Gerasymenko et al. 2019; Sheludko et al. 2018)). Bars represent standard deviations; different letters indicate significant differences at α = 0.01

Analysis of the reporter protein activity showed that the level of lichenan hydrolysis in transplastomic plants surpasses but does not differ reliably from that in hairy root cultures (1.69 ± 0.38 µmol min^−1^ mg^−1^ TSP and 0.76 ± 0.68 µmol min^−1^ mg^−1^ TSP, respectively) (Fig. 7). The reporter activity in stably transformed systems was significantly (α = 0.01) lower than the reported data of transient expression (Gerasymenko et al. 2019; Sheludko et al. 2018; Fig. 7). Western blot analysis confirmed the higher level of GFP::LicBM3 accumulation in plants transiently expressing the reporter gene as compared to the transplastomic plants (Fig. S3.9).

## Discussion

Functional heterologous expression of eukaryotic CYPs in microbial hosts is often problematic due to their dependency on the specific redox partner and the necessity of correct association with the membranes for displaying enzymatic activity. N-terminal modifications, the construction of self-sufficient enzymes possessing both P450 and redox partner domains in one polypeptide chain or co-expression of auxiliary proteins are usually necessary to achieve satisfactory yield and catalytic activity of the enzymes (Wu et al. 2021; Li et al. 2020; Hausjell et al. 2018). Plant-based expression systems offer advantages of accessibility of reducing partners and a choice of membranes to insert heterologous cytochromes. However, the available literature data do not allow for a general conclusion about the optimal subcellular compartment for the optimal exploiting of CYP enzymatic activities in plant systems.

For human CYPs, selection of the optimal localization site was carried out by transient expression of CYP2A6, CYP3A4 and CYP2D6 (Sheludko et al. 2018; Frabel et al. 2018). CYP2A6 was expressed in *N. benthamiana* as a part of biosynthetic pathway producing halogenated indican derivatives, and its catalytic activity was confirmed after localization either in the cytosol or in chloroplasts (Frabel et al. 2018). More detailed studies on transient expression of CYP2D6 and CYP3A4 in *N. benthamiana* revealed that chloroplast targeting leads to four fold decrease in activity as compared to the enzymes with the native microsomal leader sequence (Sheludko et al. 2018). It was suggested that the fusion of a chloroplast targeting peptide with a signal-anchor sequence of CYP2D6 might impair the enzyme functionality in the organelle. Recent reports also indicate a strong dependence of the activity of heterologous CYPs in chloroplasts on the molecular structure of the signal-anchor transmembrane domain and chloroplast transit peptide (de Jesus et al. 2017; Gnanasekaran et al. 2015). Loss of the activity has been reported for an ER-localized CYP720B4 of plant origin after transit to the chloroplasts. Modification of the N-terminus of CYP720B4 restored the enzyme functionality but reduced the import capacity (Gnanasekaran et al. 2015). The importance of the regions adjacent to CTP for the protein targeting was also noticed by Shen et al. (Shen et al. 2017).

In the present study, the potential problems of the transport of CYP2D6 into chloroplasts were circumvented by the expression of the gene from the plastome. We detected in transplastomic plants a high level of transcripts, surpassing manifold the levels in transiently transformed plants or hairy roots (Fig. 5A, B). However, the activities of heterologous proteins, GFP::LicBM3 reporter and CYP2D6, in transplastomic plants were significantly lower than in transiently transformed leaves (Figs. 6 and 7). A minor level of accumulation of the reporter protein in these lines was confirmed by Western blot analysis (Fig. S3.9). This discrepancy between mRNA and protein levels can be explained by mechanisms of gene expression regulation, which differ for nuclear genome and plastome. Control of translation efficiency plays a more important role in plastids (Zoschke and Bock 2018). For plastome transformation, we used the gene of interest flanked with the 5′-UTR of *psbA* operon of *M. truncatula*, which in our previous work ensured high levels of β-glucuronidase reporter activity (Gerasymenko et al. 2017). However, it is known that the translation efficiency of chloroplast transcripts may depend on the combination of 5′-UTR and the 5′-part of the coding sequence (Ancin et al. 2020; Richter et al. 2018; Farran et al. 2010; Kuroda and Maliga 2001). For example, two plant CYPs of the dhurrin metabolic pathway introduced into *N. tabacum* plastome and translated from a single polycistronic operon were accumulated in chloroplasts in concentrations differing by several orders of magnitude (Gnanasekaran et al. 2016). Therefore, utilizing an alternative 5′-UTR sequence and/or modification of the N-terminal part of CYP2D6 is necessary to improve the protein accumulation in transplastomic plants. Codon optimization of the target genes by an algorithm developed for chloroplast expression (Kwon et al. 2016) also might be beneficial for translation efficiency.

An additional factor abating enzymatic activity of CYP2D6 in chloroplasts might be an incomplete correspondence of the native microsomal leader sequence with the chloroplast translocation machinery. In the cytoplasm, the proteins equipped with an uncleavable signal-anchor sequence are inserted into the ER membrane co-translationally via the Sec61 translocation complex (Denks et al. 2014). An analogous pathway (cpSec) exists in the chloroplasts; it performs co- and post-translational transport of proteins across the thylakoid membrane. The substrates of cpSec are predominantly soluble luminal proteins and possess cleavable N-terminal thylakoid transfer sequences that resemble ER signal sequences (Fernandez 2018). The signal-anchor sequence of CYP2D6 is peculiar because it contains a cryptic mitochondrial targeting signal at its C-end. In the cytoplasm, the bimodal targeting of CYP2D6 is regulated by protein phosphorylation (Avadhani et al. 2011). It is not clear if the native N-terminus of CYP2D6 is efficient in recruiting the cpSec pathway. In addition, insertion of proteins into the thylakoid membrane mostly occurs by SRP-mediated or spontaneous mechanisms (Schunemann 2007). Chloroplast signal recognition particle (cpSRP) binds transmembrane domains and internal motifs of proteins (Ziehe et al. 2018). Nuclear-encoded single-span proteins, which are synthesized with a bipartite transit sequence including Sec-type signal peptides, can be inserted into the thylakoid membrane spontaneously (Schunemann 2007). But the effectiveness of the native N-terminus of CYP2D6 for driving this process is also not known and its optimization towards homology with thylakoid transfer sequences may facilitate the integration into the thylakoid membranes and thus improve the enzymatic activity.

One should also note that the high accumulation of a heterologous CYP with broad substrate specificity may be detrimental to plant development. In our experiments, despite the eventual formation of normal flowers and fertile seeds, transplastomic plants expressing CYP2D6 developed slower under greenhouse conditions and accumulated less photosynthetic pigments as compared to the control group transformed with a physiologically neutral reporter gene (Fig. S3.3).

As an alternative to the infiltration of plant leaves, we established stably transformed hairy root cultures expressing CYP2D6. Although the activity of CYP2D6 in this system is lower than in transiently transformed or transplastomic plants (25- and 5-fold, respectively), the feeding of substrate to hairy root cultures is more convenient, and the product processing may be facilitated by its secretion into the medium. In our experiments, hairy roots readily absorbed and accumulated more than 95% of LOR in the tissue; contrarily, a considerable part (around 20–25%) of produced DCL was secreted into the medium (Fig. S3.8). Similar data were reported for the *N. tabacum* hairy roots expressing human CYP1B1. After feeding with resveratrol, its hydroxylated derivative piceatannol was partially secreted into the culture medium. Treatment with an elicitor strongly enhanced the yield of the product and its release into the medium (Hidalgo et al. 2017). The rate of resveratrol biotransformation in hairy roots expressing CYP1B1 (up to 1.6%) was comparable to the rate of loratadine conversion in our experiments (3.73 ± 0.65%), as well as the production scale (< 1 mg l^−1^ of piceatannol in non-elicited hairy root cultures expressing CYP1B1 (Hidalgo et al. 2017) and 0.56 ± 0.10 mg l^−1^ DCL in CYP2D6 hairy roots).

Interestingly, the hairy roots of tobacco lacking heterologous *cyp* genes were capable of hydroxylation of resveratrol, though with lower efficiency (0.4%) (Hidalgo et al. 2017). We also noted the ability of hairy roots carrying the reporter gene to form DCL from LOR (1.53 ± 0.71%), as opposed to the leaves of *N. benthamiana* or *N. tabacum*. This might be the consequence of the high metabolic activity of *Nicotiana* roots, which are the site of the production of nicotine alkaloids and other secondary compounds (Zenkner et al. 2019; Pakdeechanuan et al. 2012). Multiple genes involved in biosynthesis, metabolism or transport of nicotine are expressed in tobacco roots, including members of the CYP82E subfamily encoding monooxygenases that catalyze the N-demethylation of nicotine. Though this reaction in *N. tabacum* occurs predominantly in the leaves, several homologous sequences were discovered in root-specific cDNA libraries suggesting preferential expression of these CYP genes in the root tissue (Lewis et al. 2010).

## Conclusions and future prospects

For the first time, we established transplastomic tobacco plants expressing human CYP2D6 with broad substrate specificity. The relative level of CYP2D6 transcripts in transplastomic plants was 2–3 ﻿orders of magnitude higher as compared to that observed after constitutive or transient expression from the nucleus. CYP2D6 accumulated in chloroplasts converted exogenous synthetic substrate LOR to DCL without co-expressing of the cognate CPR gene. The conversion rate (13.04 ± 3.16 mol%) in transplastomic plants surpassed three-fold that in the hairy root cultures of *N. tabacum* transformed with CYP2D6 (3.73 ± 0.65 mol%). Although the conversion of LOR in transplastomic plants is considerably lower than reported for transiently expressed CYP2D6 with the native ER signal-anchor sequence (58.06 ± 8.83 mol%), it is similar to that for the chloroplast targeted protein (17.26 ± 6.61 mol%) (Sheludko et al. 2018)).

Although currently the best strategy for implementation of CYP2D6 activity in plant system is the transient expression of ER-targeted enzyme, our results show that transplastomic plants can be a promising alternative in case of improvement of the recombinant CYP accumulation. This could be achieved by (a) selection of 5’-UTR/N-terminal signal-anchor combination for efficient translation; (b) codon optimization of the target genes by the algorithm developed for chloroplast expression (c) optimization of CYP N-terminal sequence towards homology with thylakoid transfer sequences.

Releasing the products of CYP reaction into the culture medium by hairy root cultures expressing heterologous CYPs could be of interest to continuous cultivation in a bioreactor. For this expression system, the major challenge is selecting a line with the elevated accumulation of physiologically active CYP and stimulation of the product excretion by elicitors or permeabilization agents.

## Supplementary Information

Below is the link to the electronic supplementary material.

Supplementary file1 (DOCX 3454 kb)
