# Supplementary material for: Evaluation of biotransformation capacity of transplastomic plants and hairy roots of Nicotiana tabacum expressing human cytochrome P450 2D6
Source: Transgenic Res. 2022 Apr 13;31(3):351–68. doi: 10.1007/s11248-022-00305-x (PMC9135824; doi:10.1007/s11248-022-00305-x)
Supplement: Supplementary file 1 — Supplementary file1 (DOCX 3454 kb) [file 11248_2022_305_MOESM1_ESM.docx]

Supporting Information

**TABLE OF CONTENS**

**S1 Materials and Methods 2**

**S1.1** Hairy root induction **2**

**S1.2** Extraction of loratadine (LOR) metabolites from

infiltrated leaves and isolated chloroplasts of *N. tabacum* **2**

**S2 Tables 3**

**Table S2.1** List of primer pairs employed for PCR assays  **3**

**Table S2.2** Relative transcript levels in the leaves  **4**

**S3 Figures 5**

**Figure S3.1** Schematic representation of T-DNA within the GB2.0 Ω-level

expression vector**s** **5**

**Figure S3.2** Transplastomic and control plantlets **6**

**Figure S3.3** Transplastomic (CYP2D6 and GFP::LicBM3) plants

under greenhouse conditions and content of photosynthetic

pigments  **7**

**Figure S3.4** Relative transcript levels in transgenic hairy root cultures **8**

**Figure S3.5** Structures of substrates and products of CYP2D6 reactions **9**

**Figure S3.6** HPLC-MS chromatograms of the extracts of transplastomic

*N. tabacum* plants expressing CYP2D6 **10**

**Figure S3.7** HPLC-MS chromatograms of leaf and isolated chloroplast

extracts of transplastomic *N. tabacum* plants expressing CYP2D6 **11**

**Figure S3.8** Yield of DCL in transgenic hairy root cultures of *N. tabacum*  **12**

**Figure S3.9** Western blot analysis of GFP::LicBM3 accumulation **13**

**S4. References 14**

**S1. Materials and Methods**

**S1.1 Hairy root induction**

The bacteria carrying the binary vectors were grown in the liquid YEB medium containing 100 mg/l spectinomycin and 100 μM acetosyringone at 28 °C for 48 h. The cells were harvested by centrifugation (5 min at 1000 g), resuspended in the triple volume of the liquid MS medium ([Murashige and Skoog 1962](#_ENREF_1)), supplied with a half concentration of inorganic salts and 15 g/l sucrose and 100 μM acetosyringone, and incubated at 28 °C for 1 h. The leaf explants of in vitro plants *N. tabacum* cv. Petit Havana (1x1 cm) were incubated with the suspension of *A. rhizogenes* for 5-7 min, placed onto the solid MS medium without selective agents and cultivated in the dark at 25 °C. After 96 h, the explants were rinsed three times with autoclaved water and one time with autoclaved water containing 400 mg/l cefotaxime, wiped on a filter paper and transferred onto the solid MS medium containing 450 mg/l cefotaxime. The explants were cultivated in dark at 25 °C. After 4-5 weeks, the emerging roots were detached and propagated on the solid MS medium containing 50 mg/l kanamycin. After PCR analysis confirming the presence of transgenes, the hairy root cultures were maintained on the solidified MS medium without selective agents in the dark at 25 °C with a 30–45 day period of cultivation.

**S1.2 Extraction of loratadine (LOR) metabolites from infiltrated leaves and isolated chloroplasts of *N. tabacum***

Lyophilized leaf samples (ground with a glass stick or a metal spatula) or lyophilized chloroplast preparations were extracted with 500μL of 80% (v/v) MeOH for 5 min under sonication and for further 60 min on an orbital shaker (750rpm) at 37°C. The samples were centrifuged for 5 min at 17,000×g, and the residue was re-extracted with 250μL of 80% (v/v) MeOH for 10 min under the same conditions. Extracts were combined and centrifuged, and the supernatants were applied in HPLC and HPLC–MS analyses.

**S2. Tables**

Table S1. List of primer pairs employed for PCR assays.

| Target sequence | Primer sequences (5'-3') | Amplicon size (bp) |
| --- | --- | --- |
| *rbc*L of *N. tabacum*  *aad*A | rbcLfor: aacctaaattggggttatctgc  aadArev: gagtcgatacttcggcgatcac | 1364 |
| *GFP::LicBM3*  *acc*D of *N. tabacum* | GFPfor2: aacagctcctcgcccttgc  accDrev: ctcctccgaatgaaattcaatg | 1558 |
| *CYP2D6*  *acc*D of *N. tabacum* | 2D6for2: taactgcgagtggaacgagtg  accDrev: ctcctccgaatgaaattcaatg | 1627 |
| *rbc*L of *N. tabacum*  *acc*D of *N. tabacum* | rbcLfor: aacctaaattggggttatctgc  accDrev: ctcctccgaatgaaattcaatg | 2602 (wild type);  5352 (*GFP::LicBM3*);  5475 (*CYP2D6*) |
| *rol*B | rolBfor: atggatcccaaattgctattccttccacga  rolBrev: ttaggcttctttcttcaggtttactgcagc | 776 |
| *rol*C | rolCfor: ctcctgacatcaaactcgtc  rolCrev: tgcttcgagttatgggtaca | 586 |
| *vir*D1 | virDfor: atgtcgcaaggcagtaagccca  virDrev ggagtctttcagcatggagcaa | 432 |
| *GFP::LicBM3* | GFPfor: atatcgtctcactcgaatggtgagcaagggcgaggag  GFPrev: gcgccgtctcactcgaagcttaaccgttaggatagtat | 1406 |
| *CYP2D6* | 2D6for: gcgccgtctcactcgaatgggattagaggcactcg  2D6rev: gcatcgtctcactcgaagcttatcttggcacagcac | 1529 |

**Table S2.2** Relative transcript levels in the leaves of *N. benthamiana* transiently expressing the reporter *GFP::licBM3* and *cyp2D6* genes with native microsomal leader sequence (**TE;** mean of four biological repeats for each gene; two technical repeats of each) or with N-fused CTP sequence (**TE/CTP;** mean of four biological repeats for each gene; two technical repeats of each), transplastomic T1 plants of *N. tabacum* (**TP;** the mean of five transplastomic plants two technical repeats of each) and transgenic hairy root cultures of *N. tabacum* (**HR;** the mean of four transgenic lines (*cyp2D6*) or three transgenic lines (*GFP::LicBM3*), three biological repeats of each, two technical repeats of each).

|  | **Relative expression** | |
| --- | --- | --- |
|  | Normalized by *NtIN1*, expressed in plastome (mean ± SD) | Normalized by *NtEF1α*, expressed in nucleus (mean ± SD) |
| **TE** *cyp2D6* | - | 0.88 ± 0.85 (*n* = 4) |
| **TE** *GFP::LicBM3* | - | 2.62 ± 2.40 (*n* = 4) |
| **TE/CTP** *cyp2D6* | - | 2. 051 ± 0.54 (*n* = 4) |
| **TE/CTP** *GFP::LicBM3* | - | 6.16 ± 1.82 (*n* = 4) |
| **TP** *cyp2D6* | 1190.03 ± 1060.84 (*n* = 5) | 352.44 ± 145.03 (*n* = 5) |
| **TP** *GFP::LicBM3* | 620.84 ± 268.23 (*n* = 5) | 275.64 ± 149.65 (*n* = 5) |
| **HR** *cyp2D6* | - | 0.062 ± 0.0432 (n = 4) |
| **HR** *GFP::LicBM3* | - | 0.481 ± 0.479 (n = 3) |

**S3. Figures**

A

B

C

Figure S3.1 Schematic representation of T-DNA within the GB2.0 Ω-level expression vectors. Transcriptional units include gene of interest (*GoI*, native (A) or fused to the chloroplast transit peptide sequence (CTP) (B)) under control of the CaMV 35S promoter fused to the TMV

Ω leader sequence (P35S) and the nopaline synthase terminator (Tnos), adjacent to the right border (RB), and the *p19* gene of tomato bushy stunt virus encoding a suppressor of gene silencing under control of P35S and Tnos, or the gene encoding neomycin phosphotransferase (*nptII*) (C) under control of nopaline synthase promoter (Pnos) and the CaMV 35S terminator (T35S) adjacent to the left border (LB). Constructs (A) and (B) were used for transient expression; construct (C) was applied for establishing of hairy root cultures.


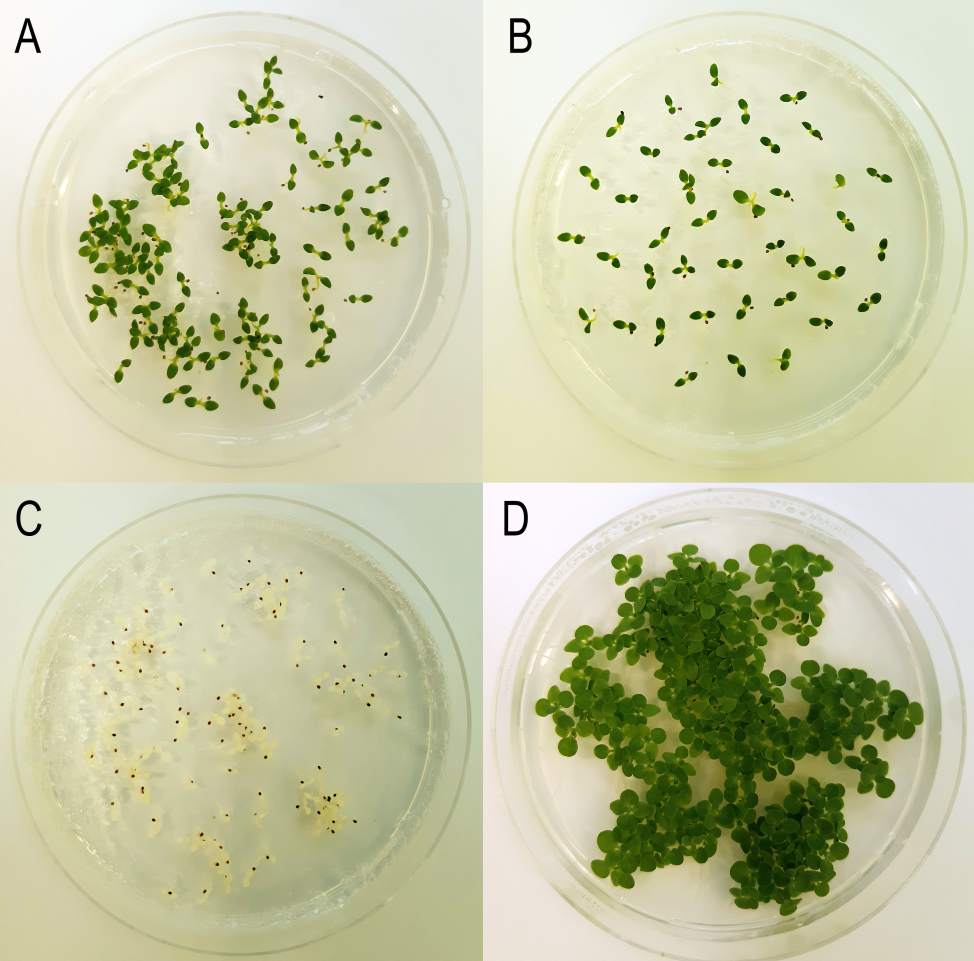


Figure S3.2 Transplastomic (GFP::LicBM3 (A) CYP2D6 (B)) (T1) and control (non-transformed wild type) plantlets (C) induced from seeds on MS medium containing spectinomycin and streptomycin (16 days after germination), and control plantlets induced from seeds on MS medium without selective antibiotics (D).


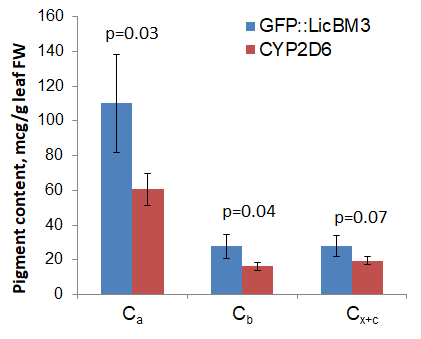

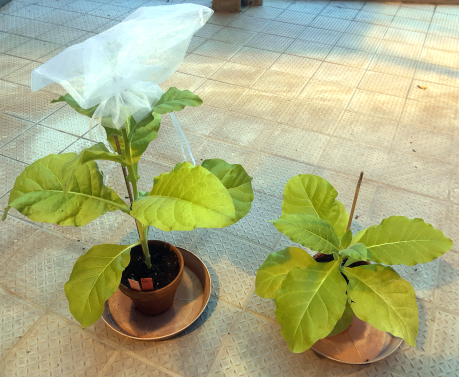


A B

Figure S3.3 (A) Transplastomic T1 plants (left: GFP::LicBM3; right: CYP2D6 ) grown in the greenhouse (26 days after transferring into soil). (B) Content of chlorophylls a and b (C_a_ and C_b_) and total carotenoids (C_x+c_) in the leaf samples (measured in 4 plants of each line in 3 technical repeats, bars represent standard deviation); p values were calculated using t-test for two samples with unequal variances.


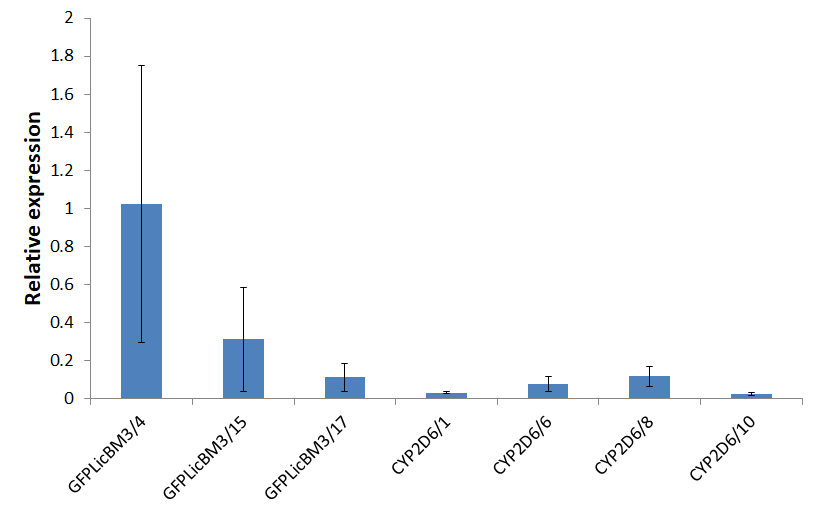


Figure S3.4 Relative transcript levels in transgenic hairy root cultures of *N. tabacum*. The gene *NtEF1α*, expressed in nucleus, was used for normalization of RT-qPCR data. The bars represent standard deviations of three biological repeats, each measured in two technical repeats.

A

B

Figure S3.5 Structures of substrates and products of CYP2D6 reactions: (A) conversion of LOR to DCL; (B) conversion of corynanthine to 11-hydroxycorynanthine ([Sheludko et al. 2018](#_ENREF_2)).


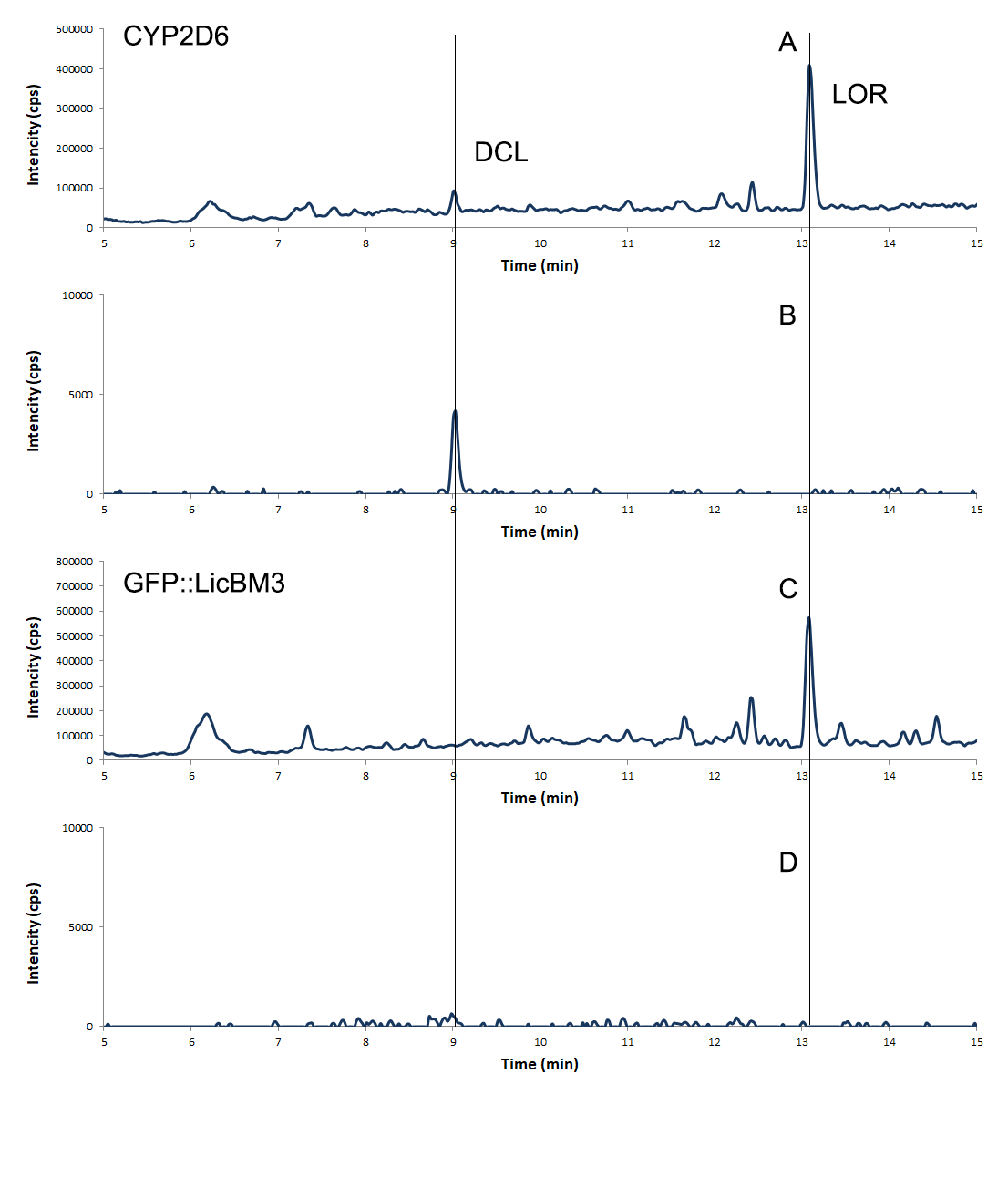


Figure S3.6 Fragments of HPLC-MS chromatograms of the extracts of transplastomic *N. tabacum* plants expressing CYP2D6. (A) Total ion chromatogram of the extract of *N. tabacum* CYP2D6 supplemented with LOR; (B) extracted ion m/z 311 chromatogram; (C) total ion chromatogram of the extract of *N. tabacum* GFP::LicBM3 supplemented with LOR; (D) extracted ion m/z 311 chromatogram.


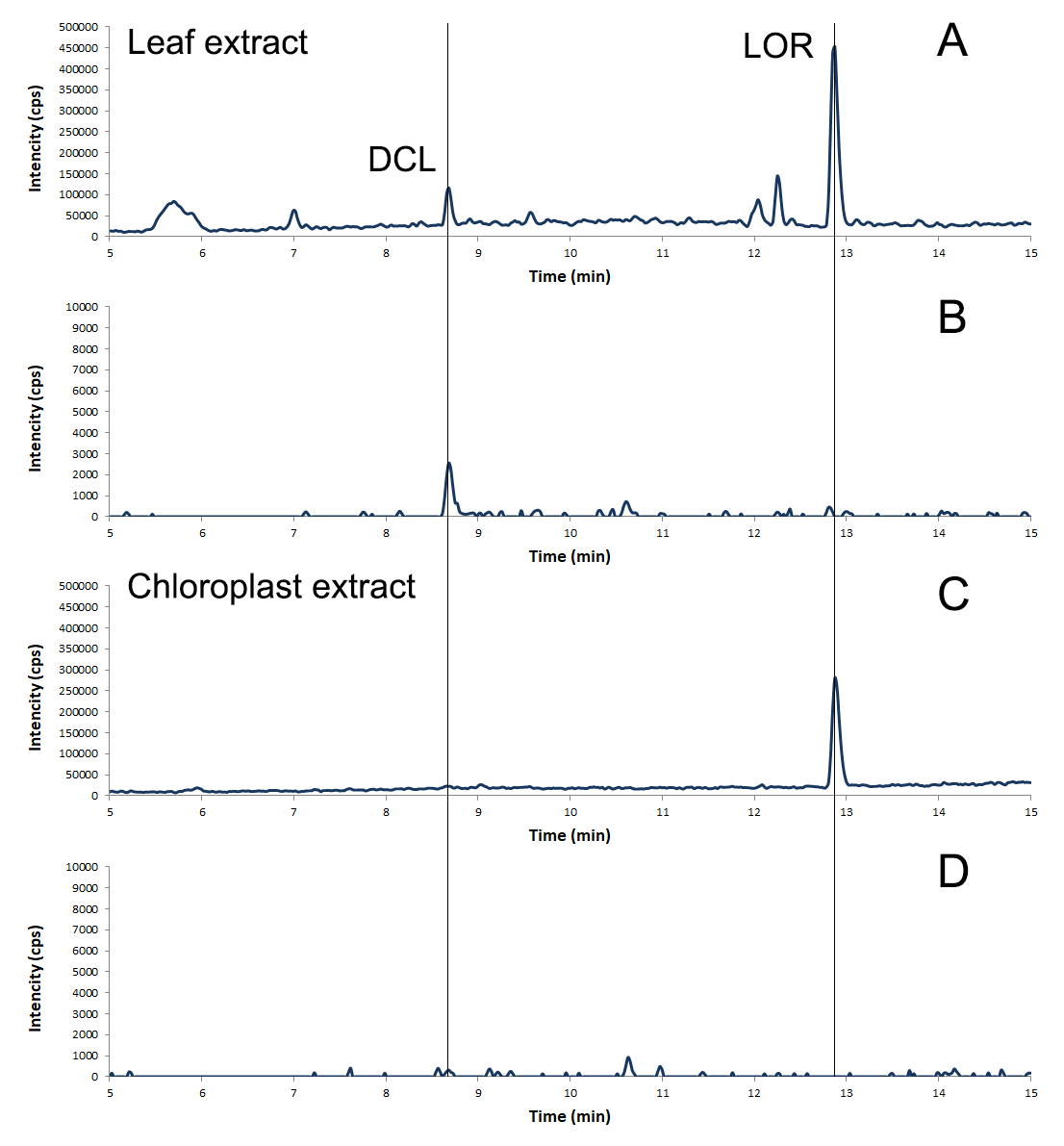
Figure S3.7 Fragments of HPLC-MS chromatograms of leaf and isolated chloroplast extracts of transplastomic *N. tabacum* plants expressing CYP2D6. (A) Total ion chromatogram of the leaf extract of *N. tabacum* CYP2D6 supplemented with LOR; (B) extracted ion m/z 313 chromatogram; (C) total ion chromatogram of the isolated chloroplast extract of *N. tabacum* CYP2D6 supplemented with LOR; (D) extracted ion m/z 313 chromatogram.


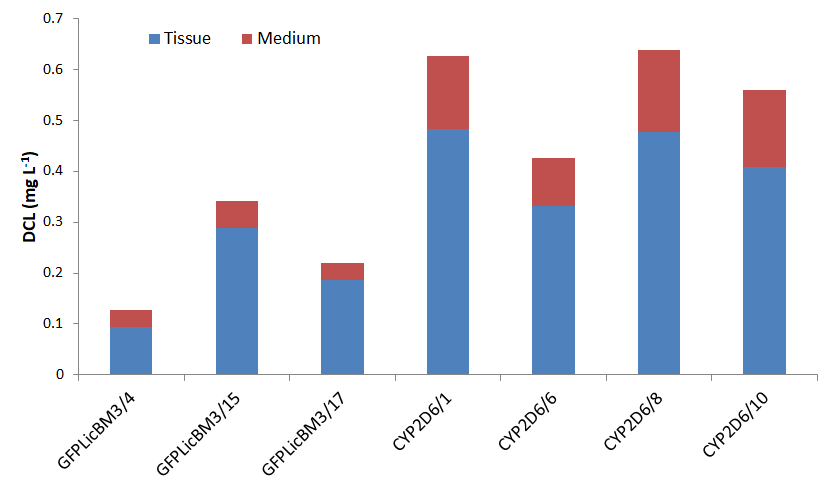


Figure S3.8 Yield of DCL in transgenic hairy root cultures of *N. tabacum* (tissue and culture medium; four transgenic lines of hairy root cultures transformed with *cyp2D6* and three transgenic lines transformed with GFP::licBM3).


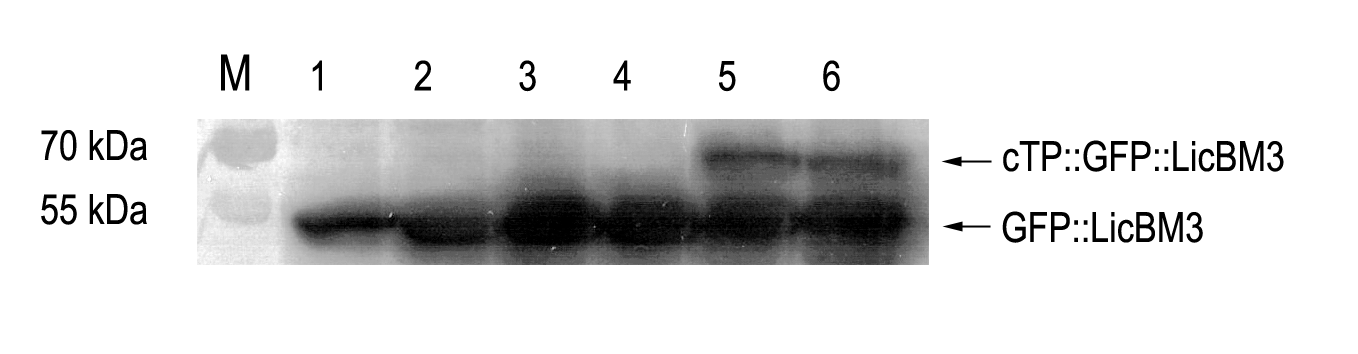


Figure S3.9 Western blot analysis of GFP::LicBM3 accumulation. Lanes 1,2: T1 transplastomic plants carrying *GFP::LicBM3* gene; lanes 3,4: plants transiently expressing *GFP::LicBM3* gene; lanes 5,6: plants transiently expressing *GFP::LicBM3* gene with the sequence encoding chloroplast targeting peptide. The band of 52 kDa observed in all plant samples corresponds to the GFP::LicBM3; the plants expressing *GFP::LicBM3* gene with the sequence encoding chloroplast transit peptide accumulate also the unprocessed protein of 58 kDa.

4. References

Murashige T, Skoog F (1962) A Revised Medium for Rapid Growth and Bio Assays with Tobacco Tissue Cultures. Physiol Plantarum 15 (3):473-497. doi:DOI 10.1111/j.1399-3054.1962.tb08052.x

Sheludko YV, Gerasymenko IM, Warzecha H (2018) Transient Expression of Human Cytochrome P450s 2D6 and 3A4 in Nicotiana benthamiana Provides a Possibility for Rapid Substrate Testing and Production of Novel Compounds. Biotechnology journal 13 (11):e1700696. doi:10.1002/biot.201700696
